# Generalized seizure: A rare etiology of intraperitoneal rupture of the urinary bladder

**DOI:** 10.4103/0970-1591.36723

**Published:** 2007

**Authors:** Bipin C. Pal, Santosh Kumar, Lalgudi N. Dorairajan, Nikhil Khattar

**Affiliations:** Department of Urology, Jawaharlal Institute of Post Graduate Medical Education and Research, Pondicherry, India

**Keywords:** Bladder injury, epilepsy, intraperitoneal bladder rupture, seizure

## Abstract

Seizures can lead to different types of injuries which can be as simple as minor lacerations and at times as serious as fractures and head injuries. We are reporting a case wherein a female patient presented with a history of abdominal pain and not passing urine for 24h following an attack of seizure. After catheterization the urine drained was blood-stained. On clinical suspicion a cystogram was done which showed intraperitoneal rupture of the bladder. At laparotomy an isolated rent in the dome of the bladder was found which was repaired in three layers. Postoperative period was uneventful. To our knowledge this is the second case of its kind reported in the literature. Our case illustrates that a thorough abdominal examination is desirable while examining a patient following an episode of generalized seizure.

## INTRODUCTION

Patients with epilepsy can suffer injuries during an attack of seizure. Most of these injuries occur during an attack of generalized tonic clonic seizures or myoclonic seizures.[1] Although various injuries have been described bladder injury has been reported extremely rarely. Our patient presented with acute abdomen after a fall during an attack of generalized tonic clonic seizure. During evaluation intraperitoneal rupture of the urinary bladder was detected which was accordingly managed. The possible mechanisms of bladder rupture due to seizures and the clinical significance of this complication are discussed.

## CASE REPORT

**Figure 1:**
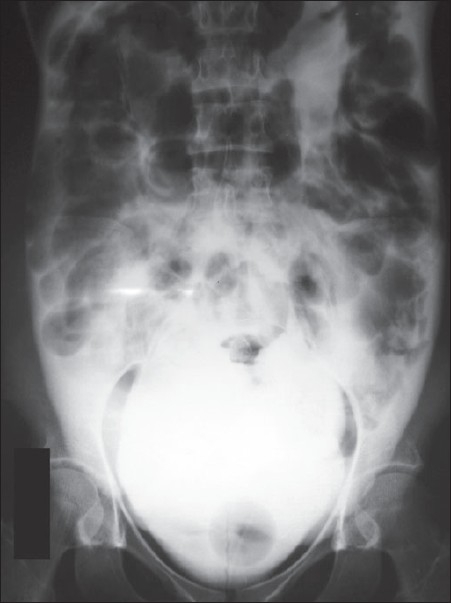
Cystogram showing contrast in the peritoneal cavity suggesting intraperitoneal rupture of the bladder

A 20-year-old female, a known seizure patient on phenytoin for six months, fell down during an attack of seizure while walking towards the toilet in the morning. She came to the emergency department after one day with complaints of pain in the abdomen and not passing urine for a day. There was no history of hematuria or any previous history of a urological disease. On examination there was no pallor. Her pulse rate was 82/min and blood pressure was 114/76mmHg. The abdomen was distended and generalized tenderness was present with suprapubic dullness extending up to the umbilicus. Bowel sounds were absent. Per vaginum and per rectal examinations were normal. She was catheterized and 1.8 liters of blood-stained urine was drained. She reported a little improvement in the abdominal pain after catheterization but the signs of peritonism persisted. Ultrasound performed after catheterization showed free fluid in the abdomen but other viscera were normal. Cystogram showed an intraperitoneal rupture of the bladder [Figure 1]. Biochemical parameters were within normal limits. Laparotomy was performed under general anesthesia. A 4cm rent present over the dome of the bladder was repaired in three layers. The bladder was drained with a suprapubic as well as a perurethral catheter. Postoperative recovery was uneventful. Her perurethral catheter was removed on Day three and the suprapubic catheter on the 10^th^ postoperative day following which she voided normally. The patient was discharged after getting a neurological consultation for long-term control of seizures.

## DISCUSSION

Seizures often lead to certain forms of injuries like scalp laceration, facial injuries, tongue bite, dental injury and even head injury of varying severity. The risk of trauma is more with generalized tonic clonic seizures and myoclonic seizures.[1,2] Patients with seizure-related trauma have significantly earlier onset age of epilepsy.[2] Seizure type, severity and frequency were the main predictors of sustaining different types of injuries.[3] A Pubmed search showed only four cases of bladder injury reported following an attack of seizure till now, one each following myoclonic and generalized tonic clonic seizure and two cases following unmodified electroconvulsive therapy.[4–6] Such an injury is extremely rare probably because the bladder usually empties during an attack of generalized tonic clonic seizures as there is loss of bladder and bowel control during the episode.

The bladder rupture occurred in our patient in the early morning when she was on her way to the toilet. We feel that the rupture occurred in our case due to sudden rise in the intraabdominal pressure when the bladder was fully distended from overnight retention. The fully distended bladder is quite thinned out and unsupported at the dome thereby predisposing to rupture at this point when there is a sudden rise in the intraabdominal pressure. In the earlier reported case by Misra *et al*., the injury occurred by a similar mechanism probably due to sudden rise of intraabdominal pressure in the background of full bladder.[4] This was attributed by them to the patient being on antipsychotic drugs which have anticholinergic properties. Two similar cases of bladder rupture have also been reported after unmodified seizures during electroconvulsive therapy in patients on medication with anticholinergic effects.[5,6] In contrast our patient was a young female not on any anticholinergic drugs. We therefore think that while such an injury may more likely occur in patients with infravesical obstruction like elderly men with benign prostatic hyperplasia or cases on anticholinergic drugs, it can occur when the patient has a fully distended bladder even in the absence of abnormal bladder voiding mechanics. Our case illustrates that physicians have to be extra careful while evaluating patients after an attack of seizure as the patient can easily forget the episode of fall or abdominal injury due to post ictal amnesia which can lead to delayed or misdiagnosis and increased morbidity and mortality. A careful abdominal examination must be performed by the physician when a patient is examined after a seizure episode.
